# Mycobacterium tuberculosis H37Rv infection regulates alternative splicing in Macrophages

**DOI:** 10.1080/21655979.2017.1387692

**Published:** 2018-02-13

**Authors:** Wei Zhang, Chen Niu, Rui-Yang Fu, Zheng-Yu Peng

**Affiliations:** a School of Medicine, Zhejiang University City College, Hangzhou, China; b MOE & MOH Key Laboratory of Medical Molecular Virology, School of Basic Medical Sciences, Fudan University, Shanghai, China; c Department of Rehabilitation, Traditional Chinese Medical Hospital of HuZhou, HuZhou, China; d Institute of Biomedical Sciences, Fudan University, Shanghai, China

**Keywords:** alternative splicing, IL4, Mycobacterium tuberculosis, SR proteins, TLR4

## Abstract

Objective: The objective of this study was to evaluate the expression of genes encoding SR proteinsand alternative splicing of IL4 and TLR4 in *Mycobacterium tuberculosis (M. tb)* H37Rv-infected macrophages.

Materials and methods: THP-1 cells were induced to differentiate into macrophages with 200 nM PMA, and H37Rv strains were used for macrophage infection. After RNA extraction, qRT-PCR was performed to evaluate the expression of many SR proteins as well as the alternative splicing of IL4 and TLR4.

Results: IL4 and TLR4 play significant roles in host immunity to tuberculosis. The level of IL-4 splice variants in THP-1 cells increased after *M. tb* H37Rv infection. Three splice variants of TLR4 were detected in *M. tb*-infected THP-1 cells, when compared with uninfected controls; the expression level of these splicing variants in *M. tb*-infected THP-1 cell was down-regulated. Since SR proteins are RNA-binding proteins that regulate RNA splicing, the expression of SR proteins was examined, and SRSF2 and SRSF3 were significantly down-regulated. In addition, splicing factors SRp75 and SF3a were also significantly down-regulated post *M. tb* infection.

Conclusion: Our findings indicate that alternative splicing may be involved in host gene regulation post *M. tb* infection of macrophage cells.

## Introduction

Approximately one-third of the world's population is estimated to be infected with *Mycobacterium tuberculosis (M. tb)*. Tuberculosis (TB) remains an important worldwide health concern, with over 9 million new cases and approximately 2 million deaths annually.^1^ Over 95% of new TB cases and deaths occur in developing countries, with the highest burden reported in Africa and Asia. *M. tb* persists inside host macrophages for a long period of time and may lead to chronic infection.^2^ A proportion of chronically infected patients may develop an active progressive infection.^3^ Unfortunately, the molecular basis that is responsible for the persistence of *M. tuberculosis* inside the host remains largely unknown,^4^ and the cause for *M. tb* reactivation is not well characterized. There is also an urgent need for *M. tuberculosis*-specific markers for diagnosis and vaccine development.

Alternative pre-mRNA splicing (AS) plays a major role in regulating gene expression and generating protein diversity.^5^ New high-throughput sequencing technology has revealed that more than 90% of human genes undergo AS, which is a much higher percentage than anticipated.^6^ Therefore, RNA splicing greatly increases the genomic complexity in higher eukaryotes. RNA splicing is processed in the spliceosome, a cellular machinery composed of five small nuclear ribonucleo-protein particles (snRNPs: U1, U2, U4/U6, and U5) and various non-snRNP proteins.^7^ Serine/arginine rich (SR) proteins, the major components of non-snRNP proteins, are critical for selective splicing and are highly regulated in various physiological and pathological conditions.^8^ RNA splicing is regulated by highly sophisticated mechanisms involving numerous RNA-binding proteins such as SR proteins and the intricate network of interactions among them. If disrupted, abnormal RNA splicing can lead to various diseases; thus, it has a profound impact on human pathogenesis.^9^

As an intracellular pathogen, *M. tb* has developed intricate strategies to evade the host surveillance system in phagocytes.^10^ One possible strategy is to disrupt alternative splicing in host cells. Recently, Haroon Kalam et al. showed extensive remodelling of alternative splicing in the macrophage transcriptome using comprehensive analysis of time-series RNA-seq data obtained from human macrophages infected with virulent or avirulent strains of *M. tb*. This finding strongly indicated that infection with *M. tb* alters the global patterns of alternate splicing within macrophages. Therefore, alternate splicing may be a new locus of intervention by *M. tb* and provides an attractive alternative to exploit for novel drug targets against *M. tb.*^11^

Additional studies are required regarding the alternative splicing event during or after the infection of M. tuberculosis. In the present study, THP-1 cells were infected with H37Rv strain, and the alternative splicing of IL-4 and TLR4 was characterized. The expression of several SR proteins/splicing factors was validated using qRT-PCR analysis.

## Materials and methods

### *M. tb* strains, cell line and infection

The reference laboratory strain H37Rv was grown at 37°C in Middle brook 7H9 broth (BD Difco), supplemented with 10% albumin-dextrose-catalase (ADC), 0.5% glycerol and 0.05% tween-80. THP-1 cell line was purchased from the cell bank of the Chinese Academy of Sciences (Shanghai, China) and cultivated at 37°C in 5% CO_2_ in RPMI 1640 (Gibco) medium supplemented with 10% FBS. Prior to infection, THP-1 cells were treated with 200 nM of PMA (Sigma) for 24 h to allow for the differentiation into macrophages in six well plates. Infections were performed with a multiplicity of infection (MOI) of 5. 7H9 broth was used as a blank control. At 18 hours post infection, cells were collected and subjected to the RNA extraction for quantitative RT-PCR analysis.

### Isolation of total RNA

After infection, cells were harvested and lysed. Cell pellets were resuspended in 1 ml of TRIzol Reagent (GIBCO) by repetitive pipetting. 100 μl of chloroform was added, and samples were centrifuged at 4,500 g for 30 min at 4°C. The colourless upper aqueous phase containing RNA was transferred to a fresh tube. The RNA was precipitated using isopropyl alcohol. The RNA pellet was rinsed with 75% ethanol. The concentration and purity of isolated RNA were determined by spectrophotometry and agarose gel electrophoresis.

### Quantitative RT-PCR (qRT-PCR) analysis

The following genes were validated by qRT-PCR: IL4-1, VIL-4, TLR4-1, TLR4-2, TLR4-3, TLR4-4, SRSF1, SRSF2, SRSF3, SRSF7, SRSF8, SRSF9, SRSF10, SRSF11, SYF2, PSF, SRp75, and SF3a. GAPDH was selected as an internal control. Primers are listed in Table S1. A total of 500 ng of RNA was reversely transcribed using SuperScript II Reverse Transcriptase (Invitrogen). The qRT-PCR was performed utilizing SYBR Green PCR Master Mix (TOYOBO) in an ABI 7900HT Sequence Detection System and analysed with SDS2.3 Software (Applied Biosystems). The number of biological replicates for all qRT-CPR experiments is three.

### Statistics

Data are expressed as the means ± SEM. Statistical significance was calculated by two-tailed t-tests. Values of p ≤ 0.05 were considered statistically significant.

## Results

### The splicing of IL-4 and TLR-4 is differentially regulated in THP-1 cells after H37Rv infection

To investigate the possible splicing genes regulated by H37Rv infection in THP-1 cells, we screened several candidates that are associated with TB infection, such as cytokines, cell receptors (TLRs, VDR), DC-SIGN, and NRAMP. We observed alternative splicing in only 2 genes, Il-4 and TLR-4.

**Figure 1.**
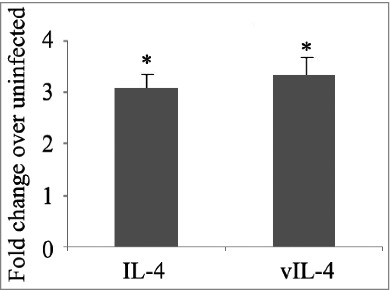
The mRNA expression of IL-4 and vIL-4 in H37Rv-infected THP-1 cells. Differences are considered to be statistically significant when P < 0.05, as indicated by stars (*), n = 4.

Human IL-4 RNA can undergo alternative splicing of exon 2. These splicing results in a naturally occurring splice variant IL-4 (VIL-4) that contains only exons 1, 3, and 4.^12^ In tuberculosis patients, it is believed that the expression level of IL-4 and its splicing variant might be associated with tuberculosis infection and chest radiographic patterns.^13^ The expression levels of IL-4 and VIL-4 in H37Rv-infected THP-1 cells were measured, and we found that both splicing variants increased post *M. tb* infection (Fig. 1). As IL-4 depletion has been shown to enhance host resistance against tuberculosis infection,^14^ the increase of both IL-4 and VIL-4 in THP-1 cells after H37Rv infection may play roles in helping *M. tb* evade the host surveillance system in phagocytes.

**Figure 2.**
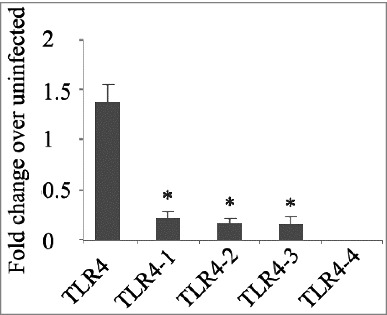
The mRNA expression of TLR-4 and splicing variants in H37Rv-infected THP-1 cells. Differences are considered to be statistically significant when P < 0.05, as indicated by stars (*), n = 4.

TLR4 has been shown to be involved in recognition of *M. tb* during infection.^15^ Whether the expression of TLR4 splice variants is relevant to *M. tb* infection is unknown. We investigated the expression of four TLR4 splicing variants in *M. tb* infected THP-1 cells. Our results showed that 3 splicing variants were similarly expressed in both control and *M. tb* infected THP-1 cells. However, the expression of TLR4 splicing variants in *M. tb* infected THP-1 cell was down-regulated (Fig. 2). Decreasing levels of TLR4 splicing variants expression may also help *M. tb* exist in macrophage by reducing recognition.

### The expression of SR proteins and splicing factors in THP-1 cells are inhibited post H37Rv infection

**Figure 3.**
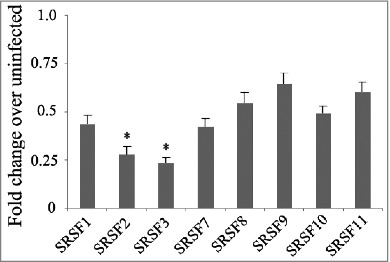
The mRNA expression of SR proteins in H37Rv-infected THP-1 cells. Differences are considered to be statistically significant when P < 0.05, as indicated by stars (*), n = 4.

**Figure 4.**
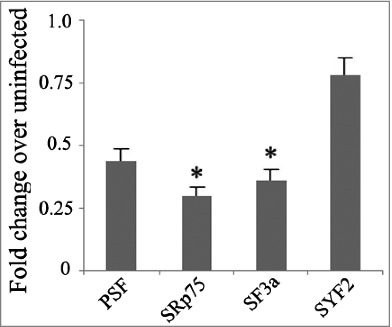
The mRNA expression of splicing factors in H37Rv-infected THP-1 cells. Differences are considered to be statistically significant when P < 0.05, as indicated by stars (*), n = 4.

Serine/arginine rich (SR) proteins are critical for selective splicing and are highly regulated in various physiologic and pathologic conditions.^16^ Few studies have examined SR proteins in *M. tb* infection. However, Danelishvili et al. reported that the polypyrimidine tract binding protein-associated splicing factor (PSF) can be recognized and cleaved by Rv3654c.^17^ Here, we examined the expression of a series of SR proteins (SRSF1, SRSF2, SRSF3, SRSF7, SRSF8, SRSF9, SRSF10, and SRSF11) and splicing factors (SYF2, PSF, SRp75, and SF3a) in THP-1 cells post *M. tb* infection. We found that the expression levels of SRSF1, SRSF7, SRSF8, SRSF9, SRSF10, and SRSF11 did not show any significant changes in regulation, but the expression levels of SRSF2 and SRSF3 were significantly down-regulated. The splicing factors SRp75 and SF3a were also significantly down-regulated (Fig. 3, Fig. 4).

## Discussion

The complex interaction between host RNA splicing machineries and viral components has been studied intensively in the last decade. Viral proteins regulate the splicing of pre-mRNAs by interacting with host RNA splicing machinery.^18^ Numerous immunologically relevant genes undergo alternative splicing,^19^ but it is still unclear whether bacteria could affect alternative splicing to deregulate the host immune responses. A recent study that analysed the changes in the phosphoproteome of gastric cells upon *Helicobacter pylori* infection, reported an enrichment of RNA processing and splicing factors in infected cells.^20^ Okuda et al. found that *Shigella flexneri* protein IpaH9.8 binds to the splicing factor U2AF to modulate host immune responses.^21^ During *M. tb* infection, it has been shown that Rv3654c recognizes the polypyrimidine tract-binding protein-associated splicing factor (PSF) and cleaves it, thus diminishing the availability of caspase-8, thus indicating a role of alternative splicing in the suppression of macrophage apoptosis after *M. tb* infection.^17^ In addition to this, evidence of the association between splicing and mycobacterial infection is lacking.

IL-4 has an implicated role in the conversion of LTB to active TB. Studies by Rook et al. have demonstrated that patients with TB have a higher level of VIL-4, a splice variant of IL-4, which was associated with the more-severe TB-induced lung damage.^22,23^ Consistent with this finding, our study demonstrates that both IL-4 and VIL-4 were increased significantly in THP-1 cells post *M. tb* infection. Previous results have suggested that VIL-4 may be an antagonist to IL-4-induced cell proliferation and the expression of CD23.^24^ However, further studies are needed to clarify the function and corresponding mechanism of VIL-4.

Toll-like receptors (TLRs) are expressed on the surface of the cell membrane or on the membrane of endocytic vesicles of major immune cells including macrophages and dendritic cells (DCs). TLR4 is known to be involved in the recognition of *M. tb*, and can activate NF-κB via signal transduction, which is involved in various intracellular signalling systems.^25–27^ For alternative splicing of human TLR4, it is known that exon II and exon III are alternatively spliced, thus producing four splice variants.^28^ The function of these splice variants is unknown. We report here that the expression levels of TLR4 and three variants in *M. tb*-infected THP-1 cells was decreased compared with uninfected control. Such a decrease may play important roles during *M. tb* infection. However, as studies on the role of TLR4 in the recognition of *M. tb* have shown conflicting results, further studies are necessary to elucidate the function of these splicing variants and the role of TLR4 for *M. tb* infection.

Haroon Kalam et al. studied the splicing of the components of the spliceosome complex post *M. tb* infection and found that, of nearly 131 genes involved in splicing, the majority did not show any significant regulation. This finding indicated that pre-mRNA splicing of SR genes themselves were not crucial for *M. tb* infection.^11^ However, it was shown from their data that IL4 was not expressed after infection and TLR4 decreased after infection. Additionally, they did not detect significant IL4 and TLR4 splicing variants. This difference might be due to different time points post infection when samples were collected. As the main regulator of pre-mRNA splicing, RNA expression of a series of SR genes and splicing factors was detected by real-time PCR, and we found that *M. tb* infection significantly down-regulated the expression levels of SRSF2, SRSF3, SRp75 and SF3a. Previously, PSF has been shown to be down-regulated during *M. tb* infection, and it participates in preventing the apoptosis of *M. tb* infected macrophages.^17^ However, the roles of SRSF2, SRSF3, SRp75 and SF3a in *M. tb* infection need to be elucidated in future studies. As SR proteins are required in both constitutive and alternative splicing of host cells and play important roles in normal biological processes, suppressing the expression of SR proteins/splicing factors could be a strategy for *M. tb* to escape killing by macrophages. The interplay between viral and host SR proteins in the regulation of both viral and host gene expression has been intensively studied.^18^ Through the RS domain or RS-rich motifs present in viral proteins, viruses may efficiently exploit the host splicing machineries to benefit their own gene expression. Moreover, through interactions with host SR proteins or by modifying their phosphorylation status, several non-SR viral proteins may interfere with cellular gene expression at the post-transcriptional level. To this end, future studies are needed to address several interesting questions: 1) Why does *M. tb* infection down-regulate the expression of SR proteins/splicing factors? 2) Can this bacillus directly interact with cellular SR proteins or splicing factors? 3) Does this bacillus modify phosphorylation status of SR proteins or splicing factors? These studies will allow for a more comprehensive understanding of alternative splicing governed by *M. tb* and will facilitate the future development of anti-tuberculosis strategies.
